# Drug Use Disorders and Violence: Associations With Individual Drug Categories

**DOI:** 10.1093/epirev/mxaa006

**Published:** 2020-10-02

**Authors:** Shaoling Zhong, Rongqin Yu, Seena Fazel

**Keywords:** crime, meta-analysis, opioid, sedative, stimulant, substance misuse, substance use disorder, violence

## Abstract

We conducted a systematic review that examined the link between individual drug categories and violent outcomes. We searched for primary case-control and cohort investigations that reported risk of violence against others among individuals diagnosed with drug use disorders using validated clinical criteria, following Preferred Reporting Items for Systematic Reviews and Meta-Analysis guidelines. We identified 18 studies published during 1990–2019, reporting data from 591,411 individuals with drug use disorders. We reported odds ratios of the violence risk in different categories of drug use disorders compared with those without. We found odds ratios ranging from 0.8 to 25.0 for most individual drug categories, with generally higher odds ratios among individuals with polydrug use disorders. In addition, we explored sources of between-study heterogeneity by subgroup and meta-regression analyses. Cohort investigations reported a lower risk of violence than case-control reports (odds ratio =  2.7 (95% confidence interval (CI): 2.1, 3.5) vs. 6.6 (95% CI: 5.1, 8.6)), and associations were stronger when the outcome was any violence rather than intimate partner violence (odds ratio = 5.7 (95% CI: 3.8, 8.6) vs. 1.7 (95% CI: 1.4, 2.1)), which was consistent with results from the meta-regression. Overall, these findings highlight the potential impact of preventing and treating drug use disorders on reducing violence risk and associated morbidities.

## Abbreviations

CI: confidence interval
OR: odds ratio

## INTRODUCTION

Drug misuse is a global public health concern (1, 2). Worldwide, around 70 million individuals were diagnosed with a drug use disorder (1). Drug use disorders have been associated with a wide range of adverse outcomes, including suicide, comorbid mental illness, and premature mortality (3–5). In addition, drug use disorders increase risk of violence against others (3, 6–9). Further, the prevalence of drug use disorder in prison ranges from 10% to 61% in men and 30% to 69% in women (10), which is substantially elevated compared with the prevalence, ranging from 0.6% to 4.0% in men and 0.3% to 2.9% in women, in the general population (11).

The prevalence differs between individual categories of drug use disorders. Globally, the prevalence rate per 100,000 people is 65 for stimulants such as amphetamines, 78 for cocaine, 290 for cannabis, 353 for opioids, and less than 52 for other drugs including hallucinogens and sedatives (12). Although research has consistently found increased violence risk in drug use disorders, individual studies have shown that the magnitude of this increased risk varies depending on the drug category. For example, when compared with the general population, odds ratios of violence in cannabis use disorder have ranged from 1 to 7 (13–17), and in cocaine, they have varied from 2 to 11 (18–21). This might be due to different methodologies adopted and specific outcomes used in different studies. Furthermore, it has been suggested that certain type of stimulants, such as crack cocaine, that are associated with irritability and aggressiveness (7, 22), might have a higher risk of criminal behavior than others, including less-strong forms of cannabis that might reduce risks due to sedative and calming effects (23, 24). This is important to clarify further in that more precise estimates would allow for risk stratification, better treatment allocation (especially if liaison with criminal justice agencies is required), and more evidence-based estimates of the population impact of certain drug policies. 

Previous reviews have explored associations between general drug misuse and violence against others but have mostly investigated selected samples, such as prisoners (25) or psychiatric patients (26–29). In addition, most existing reviews have not used standardized clinical criteria to identify drug use disorders (22, 30). This could introduce bias given that self-report of the extent of drug use is often unreliable (31). Validated diagnostic tools based on validated criteria (such as the *Diagnostic and Statistical Manual of Mental Disorders* or *International Classification of Diseases*) can identify individuals with a severe form of drug misuse, who might present to clinical and addiction services, and for whom there is evidence-based treatment available. In addition, diagnostic categories enable consistent communication between clinicians and researchers because the criteria are widely known and validated cross-culturally with decent reliability measures (32, 33). Furthermore, the most recent review that examined the link between general drug use disorders and violence was conducted more than 2 decades ago (34) and did not explore potential source of between-study heterogeneity or differences between individual categories of druguse.

The link between drug use and violent outcomes is complex; a wide range of factors—such as experiences of violence including both as victim and perpetrator, the comorbidity of other mental disorders, and social determinants such as sex, ethnicity, and poverty—might moderate and mediate this link. For instance, previous violence victimization might trigger development of drug use disorders, which might in turn lead to later perpetration of violence (35–39). Moreover, structural causes of drug use problems are relevant, given that they have been linked to criminalization (23), as well as factors such as poverty (40), poor mental health (4, 41), treatment availability (42), and homelessness (43). In addition, physical and psychological effects of drugs can lead to agitation, aggression, and cognitive impairment that might in turn heighten risk of violence. Individuals with drug use disorders might also turn to violence to finance their drug use, and disputes within illegal drug markets might be associated with violence (44). To address these gaps in the evidence, in this review, we aimed to synthesize the odds of violence in individual drug use disorders and explore sources of heterogeneity between studies.

**Figure 1:**
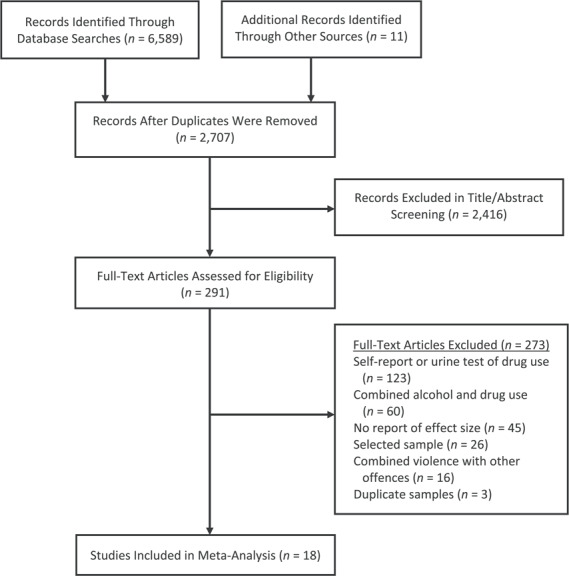
Flowchart of systematic search strategy of primary studies on drug use disorders and violence in multiple countries, 1990–2019.

## METHODS

We conducted this review following the Meta-analyses of Observational Studies in Epidemiology (MOOSE) (45) and Preferred Reporting Items for Systematic Reviews and Meta-Analysis (PRISMA) guidelines (46). The study was registered with an international prospective register of systematic reviews (PROSPERO CRD42019119533).

### Search strategy

We conducted searches in the following digital databases from the inception of the databases (dated from January 1, 1927) to February 18, 2019: PubMed, Web of Science, Embase, Ovid MEDLINE, PsycINFO, Global Health, and US National Criminal Justice Reference Service Abstract Database. We used a combination of search terms related to drug misuse (i.e., illegal drug OR illegal substance OR marijuana OR cocaine OR cannabis OR opioid OR heroin OR methamphetamine OR stimulant*) AND violence (i.e., violen* OR crim* OR homicide OR aggress* OR offen*) AND study design (i.e., cohort OR longitudinal OR follow-up OR prospective OR case-control). We included studies of both illegally and legally obtained drugs. There were no language restrictions, and non-English-language articles were translated. We also scanned reference lists in an attempt to identify additional articles. We searched for unpublished literature including conference proceedings, theses, and dissertations. The first author (S.Z.) conducted the initial screening of the titles and abstracts for inclusion and exclusion. S.Z. and R.Y. screened full-text publications for eligibility. Any uncertainties were discussed with S.F.

### Study selection

Inclusion criteria were: 1) cohort and case-control studies that examined link between individual categories of drug use disorders and violent outcomes and provided data for calculation of odds ratio between individuals with and without the drug use disorder being studied, and eligible case-control studies were those that reported prevalence of drug use disorders in cases with and without violence perpetration; 2) investigations that reported drug use disorders (or, in older studies, equivalent diagnostic categories of drug abuse or dependence) meeting diagnostic criteria for *Diagnostic and Statistical Manual of Mental Disorders* and *International Classification of Diseases*; and 3) studies that reported violent outcomes, including any violence and not being limited to context (e.g., community, domestic, intimate partner), type of crime (e.g., homicide, assault, threat or intimidation, and all sexual offenses), and measures (self-report, family report, or official/criminal records).

We excluded: 1) animal investigations; 2) experimental, cross-sectional, qualitative studies, or randomized controlled trials; 3) investigations with within-individual designs; 4) studies that used self-report (47) (e.g., Addiction Severity Index) or urine tests to identify drug use or that did not separate drug misuse from alcohol and nicotine misuse; 5) reports with recidivism or reoffending as outcomes (48); 6) studies in selected samples (e.g., offenders, cohorts with mental disorders) so we could increase the generalizability of risk estimates to the general population; 7) investigations that used the nonspecific outcome of all criminal behavior, antisocial behavior, or delinquency, which was not broken down for violence specifically; 8) studies that reported selected participants under medication (e.g., antidepressant, antipsychotic drugs, or other prescription drugs) or individuals undergoing other interventions for drug use disorders; or 9) case-series studies or reviews. 

In case of duplicate samples, we included the study that was most recent, used the most common outcome, or had the largest sample. If a study reported outcomes at multiple time points, outcomes with the longest follow-up period were included.

### Data extraction

We used a standardized form to extract data. The following information was recorded: study design, country, sample characteristics, diagnostic criteria, category of drug use disorders, type of drugs, comparison group, sex, age, years of follow-up, and study period. S.Z. conducted the initial data extraction. In case of uncertainties, R.Y. and S.F. were consulted.

### Statistical analysis

Quality of the individual study was assessed using the Newcastle-Ottawa Quality Assessment Scale (49). Heterogeneity was estimated using *I*^2^. *I*^2^ is reported as a percentage out of 100%, where 0%–40% represents low heterogeneity, 30%–60% might indicate moderate heterogeneity, 50%–90% might denote substantial heterogeneity, and 75%–100% might indicate considerable heterogeneity (50, 51). All effect sizes were converted into odds ratios and converted from Pearson’s *r* and Cohen’s *d* using standard approaches (52). Sources of heterogeneity were explored using subgroup analyses and meta-regression analyses. Meta-regression was conducted to estimate the extent to which one or more measured covariates (the same variables as used in the subgroup analysis) explained the observed heterogeneity in risk estimates between primary studies (50). The same variables were used in the subgroup and meta-regression analyses, and only nonoverlapping samples were included in the analyses. When testing the effect of sample size, we excluded 2 studies that were disproportionately large (53, 54). We set the years of follow-up as a continuous variable and also a dichotomous variable using the median period as the cut-off. Other analyses included estimating associations between drug use disorders and violence while excluding studies published before 2000 and subgroup analyses by different comparison groups. We tested publication bias using Egger’s test (55), with *P* < 0.05 indicating publication bias. Analyses were performed using STATA, version 13 (StataCorp LP, College Station, Texas).

## RESULTS

We identified 18 eligible studies (for details, see Figure 1 and Table 1) that included 591,411 individuals with drug use disorders. Studies were from 5 countries: 14 from the United States (*n* = 542,393, 91.7%) (53, 54, 56–67) and 1 each from New Zealand (*n* = 182, 0.03%) (68), Denmark (*n* = 43,403, 7.3%) (69), the Netherlands (*n* = 5,303, 0.9%) (70), and Turkey (*n* = 130, 0.02%) (71). Eight studies used case–control designs (53, 54, 56, 58, 63, 66, 67, 71); the remaining 10 studies were longitudinal cohorts with a median follow-up of 9.5 years.

**Table 1:** Summary of Included Studies on Risk of Violence in Drug Use Disorders

**First Author, Year (Reference No.)**	**Country**	**Source of Population**	**DiagnosisCriteria**	**Design**	**Type of Drugs**	**ComparisonGroups** ^**a**^	**Sample Size**	**Age, years**	**Sex**	**Year**	**Follow-up, years**	**Outcome**	**Source of Outcome**	**Assessed Alcohol Use**
Swanson, 1990 (56)	US	The Epidemiologic Catchment Area surveys	DSM-III^a^	Case-control	Cannabis use dependence, other drugs	Non–drug use disorder	8,061	≥18	Mixed	1983	N/A	Violence	Self-report	Yes
Friedman, 1996 (57)	US	A longitudinal study of the National Collaborative Perinatal Project	DSM-III	Cohort	Marijuana use, drug abuse	Non– marijuana use, non–drug abuse	380	25.5 ± 6.1	Mixed	1985	2.5	Violence	Self-report	Yes
Arseneault, 2000 (68)	New Zealand	The Dunedin Study	DSM-III-R	Cohort	Marijuana dependence disorder	Non–marijuana dependence disorder	182	Mean, 21	Mixed	1994	21	Violence	Court convictions and/or self-report	Yes
Corrigan, 2005 (66)	US	The National Comorbidity Survey (NCS)	DSM-III-R	Case-control	Any drug use disorder	Non–drug use	5,865	18–54	18–54	1990–1992	N/A	Violent behavior	Self-report	Yes
Payer, 2011 (58)	US	Participants diagnosed with methamphetamine dependence	DSM-IV	Case-control	Methamphetmine dependence	Healthy controls without drug use disorder	44	32.8 ± 8.8	Female	Not stated	N/A	Aggression	Self-report	Yes
Christoffersen, 2003 (69)	Denmark	The 1966 birth cohort in Denmark	ICD-8^b^	Cohort	Drug addicts	Non–drug addicts	43,403	15–47	Male	1993	13	Violent crimes	Official records	No
Feingold, 2008 (59)	US	The Couple Study associated with the Oregon Youth Study	DSM-IV	Cohort	Cannabis, hallucinogen, cocaine, opiates, amphetamines, sedatives	Non–cannabis, hallucinogen, cocaine, opiates, amphetamines, sedatives	150	19–28	Male	Not stated	9	Intimate partner violence	Self-report, other’s report, interview ratings	Yes
Van Dorn, 2012 (60)	US	National Epidemiologic Survey on Alcohol and Related Conditions	DSM-IV	Cohort	Drug use disorder	Non–drug use disorder	36,019	≥18	Mixed	2005	3	Any violence	Self-report	Yes
Smith, 2014 (61)	US	National Epidemiologic Survey on Alcohol and Related Conditions	DSM-IV	Cohort	Cocaine, cannabis, opioid use disorder	Non–cocaine use disorder, non–opioid use disorder	25,633	Mean, 46.4	Mixed	2005	3	Intimate partner violence	Self-report	Yes
Feingold, 2014 (62)	US	The Couple Study associated with the Oregon Youth Study	DSM-IV-TR	Cohort	Cannabis, hallucinogen, cocaine, opiates, amphetamines, sedatives	Non–cannabis, hallucinogen, cocaine, opiates, amphetamines, sedtives	146	Mean, 35	Female	Not stated	10	Intimate partner violence	Others’ report	Yes
Have, 2014 (70)	Netherlands	A cohort study of the Dutch general population	DSM-IV	Cohort	Drug dependence	Non–drug dependence	5,303	18–64	Mixed	2012	3	Physical violence	Self-report	Yes
McCauley, 2015 (63)	US	The National Comorbidity Survey Replication	WHO-CIDI^c^	Case-control	Drug abuse	Non–drug abuse	5,692	21–99	Mixed	2003	N/A	Physical dating violence	Self-report	Yes
Harford, 2016 (53)	US	National Survey on Drug Use and Health	DSM-IV	Case-control	Drug use disorder	Non–drug use disorder	108,560	12–17	Mixed	2008–2013	N/A	Other-directed violence	Self-report	Yes
White, 2015 (64)	US	A cohort of the Pittsburgh Youth Study	DSM-IV	Cohort	Cannabis use disorder, hard drug use disorder	No substance use disorder	240	35.8 ± 0.8	Male	2010	10	Persist in violence	Self-reports and official charges	Yes
Trauffer, 2017 (65)	US	A cohort study in which children were followed into adulthood	DSM-III-R	Cohort	Drug abuse and, or dependence	Non–drug abuse/dependence	413	29.6± 3.9	Female	2014	28	Violent offender	Official records	Yes
Harford, 2018 (54)	US	National Survey on Drug Use and Health	DSM-IV	Case-control	Any drug use disorder	Non–drug use	314,881	≥18	Mixed	2008–2015	N/A	Other-directed violence	Self-report	Yes
Harford, 2018 (67)	US	The National Epidemiologic Survey on Alcohol Related Conditions-III	DSM-V	Case-control	Cannabis, opioid, other drug use disorder	Non–drug use	36,309	≥18	Mixed	2012–2013	N/A	Other-directed violence	Self-report	Yes
Altintas, 2019 (71)	Turkey	Outpatients	DSM-IV	Case-control	Synthetic cannabinoid use disorders	Healthy volunteers without substance use disorders	130	28.2 ± 7.6	Mixed	Not stated	N/A	Aggression	Self-report	No

Abbreviations: DSM, *Diagnostic and Statistical Manual of Mental Disorders*; ICD, *International Classification of Diseases*; N/A, not available; WHO-CIDI; World Health Organization’s Composite International Diagnostic Interview.

^a^ Non–drug, non–drug use disorder, and non–drug addicts served as a control group and they refer to people who might have used drugs before but did not meet diagnostic criteria for any drug use disorders. “Healthy volunteers without substance use disorders” refers to healthy volunteers without drug or alcohol use disorders.

In 16 investigations, diagnosis was made using the *Diagnostic and Statistical Manual of Mental Disorders* (version 3 onward). One study adopted the *International Classification of Diseases*, *Eighth Revision* (69), and 1 provided both *International Classification of Diseases*, *Tenth Revision*, and *Diagnostic and Statistical Manual of Mental Disorders–IV* diagnoses (63).

For outcome measurement, 2 studies used violent conviction from official records (65, 69) and 1 reported intimate partner violence from the partner’s report (62). Most used self-report items in the Diagnostic Interview Schedule (56), PPC Delinquency and Criminal Behavior inventory (57), Aggression Questionnaire (58), Conflict Tactics Scale (63), physical aggression subscale in Buss-Perry Scale (71), and specially developed questionnaires (53, 54, 60, 61, 66, 67, 70). A combination of several measures (e.g., official records and self-report) was applied in 3 studies (59, 64, 68).

### Any drug or polydrug use disorder

**Figure 2:**
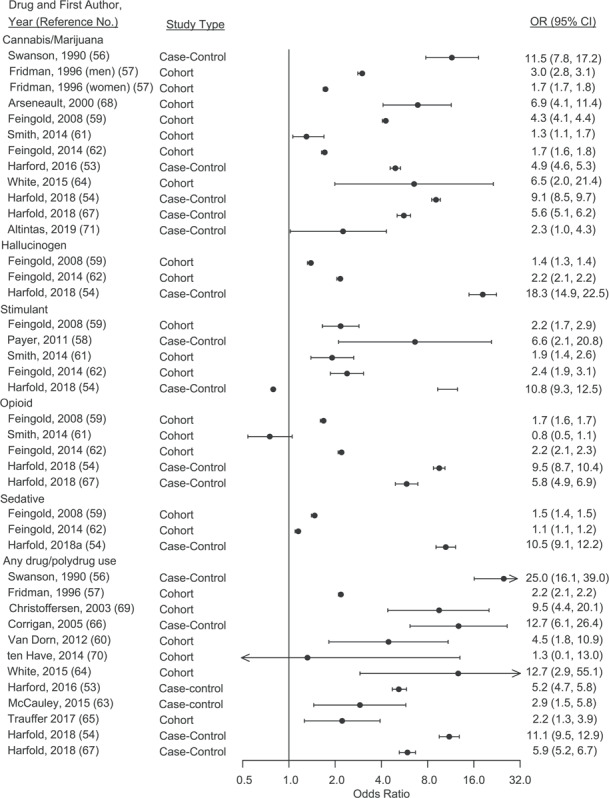
Odds ratios (ORs) and confidence interval (CIs) for the association between drug use disorders and violent outcome. Weights are from random effects analysis.

We identified 6 cohort investigations (57, 60, 64, 65, 69, 70) and 6 case-control reports (53, 54, 56, 63, 66, 67) that examined the risk of violence in any or polydrug use disorder. The odds ratios ranged from 1.3 (95% confidence interval (CI): 0.1, 13.0) to 25.0 (95% CI: 16.1–39.0) (Figure 2). When excluding the 2 studies that were published prior to 2000, the odds ratio was 4.1 (95% CI: 3.0, 5.7).

### Cannabis/marijuana use disorder

Six cohort studies (57, 59, 61, 62, 64, 68) and 5 case-control investigations (53, 54, 56, 67, 71) examined the link between cannabis/marijuana use disorder and violence. The odds ratios ranged from 1.3 (95% CI: 1.1, 1.7) to 11.5 (95% CI: 7.8, 17.2). When excluding studies prior to 2000, the odds ratios ranged from 1.3 (95% CI: 1.1, 1.7) to 9.1 (95% CI: 8.5, 9.7). (See Figure 2).

### Hallucinogen use disorder

Two cohort investigations (59, 62) and 1 case-control report (54) tested the association between hallucinogen use disorder and violence. The odds ratios varied from 1.4 (95% CI: 1.3, 1.4) to 18.3 (95% CI: 14.9, 22.5). (See Figure 2).

### Stimulant use disorder

We identified 5 studies that reported risk estimates for violence in stimulant use disorder, with 3 studies (59, 61, 62) using a cohort study design and 2 (54, 58) using a case-control study design. All of these studies were conducted in the United States. The odds ratios ranged from 1.9 (95% CI: 1.4, 2.6) to 10.8 (95% CI: 9.3, 12.5). (See Figure 2).

### Opioid use disorder

Three cohort investigations (59, 61, 62) and 2 case-control studies (54, 67) reported the risk of violence in opioid use disorder, all of which were conducted in the United States. The risk estimates ranged from an odds ratio of 0.8 (95% CI: 0.5, 1.1) to 9.5 (95% CI: 8.7, 10.4). (See Figure 2).

### Sedative use disorder

Two cohort investigations (59, 62) and 1 case-control study (54) examined the association between sedative use disorder and violence. Odds ratios varied from 1.1 (95% CI: 1.1, 1.2) to 10.5 (95% CI: 9.1, 12.2). (See Figure 2).

### Heterogeneity

No significant differences were found in risk estimates by sex, country, outcome measures, years of follow-up, and sample size in subgroup analyses (Table 2). The risk estimates in cohort investigations (odds ratio (OR) = 2.7, 95% CI: 2.1, 3.5) were lower than in the case-control reports (OR = 6.6, 95% CI: 5.1, 8.6). No differences were found among violence by self-report (OR = 4.6, 95% CI: 3.0, 7.2), informant report/official records (OR = 3.2, 95% CI: 1.3, 7.8), and combined measures (OR = 4.4, 95% CI: 1.3, 14.5).

**Table 2:** Risk Estimates for Violence in Drug Use Disorders According to Sample or Study Characteristics, Multiple Countries, 1990–2019

**Source of Heterogeneity**	**No. of Studies**	**No. in Population**	**OR**	**95% CI**
Sex				
Male	4	43,976	3.9	1.7, 8.9
Female	4	800	2.2	1.8, 2.7
Mixed	11	546,635	5.4	4.1, 7.0
Study location				
United States	14	542,393	4.2	2.9, 6.1
Other high-income counties	3	48,888	7.1	4.1, 12.2
US study locations				
National	8	533,339	4.4	2.9, 6.1
Region-based	6	9,054	3.9	2.0, 7.6
Measures of outcome				
Self-reported outcome	12	546,877	4.6	3.0, 7.2
Others’ report/official records	3	43,962	3.2	1.3, 7.8
Combined measures	3	572	4.4	1.3, 14.5
Temporality in cohort studies				
Drug prior to violence	4	85,105	3.8	1.6, 9.1
Others	6	26,764	2.6	1.6, 4.3
Study design				
Cohort study	10	111,869	2.7	2.1, 3.5
Case-control study	8	473,850	6.6	5.1, 8.6
Years of follow-up				
<9.5 years	5	67485	1.9	1.5, 2.4
≥9.5 years	5	44384	4.5	2.2, 9.1
sample size				
<500	8	1503	2.6	2.0, 3.4
≥500	8	166285	5.1	2.9, 9.2
Violent outcome				
Intimate partner violence	4	31,621	1.7	1.4, 2.1
Intimate partner violence with general controls	2	31,325	1.8	0.8, 4.2
General violence	14	559,790	5.7	3.8, 8.6
Clinical criteria				
DSM-III (Revision)	5	14,901	5.7	2.5, 13.0
DSM-IV (Text Revision)	10	491,106	3.4	2.0, 6.0

Abbreviations: CI, confidence interval; DSM, *Diagnostic and Statistical Manual of Mental Disorders*; OR, odds ratio.

The odds ratios for intimate partner violence (OR = 1.7, 95% CI: 1.4, 2.1) were lower than for general violence (OR = 5.7, 95% CI: 3.8, 8.6) (Table 2). When further exploring the associations of the comparison groups in studies of the association between drug use disorders and intimate partner violence, no significant differences were found. In the meta-regression analysis, we found that study design (cohort vs. case–control study) was associated with heterogeneity ($\beta $ = 0.8, *t* = 2.3, *P* = 0.04), as was the violent outcome (intimate partner violence vs. general violence; $\beta $  = −1.2, *t* = −3.3, *P* = 0.004). No other variables examined explained the heterogeneity between studies. Egger’s test did not suggest publication bias (*t* = 1.32, *P* = 0.20).

## DISCUSSION

### Main findings

This systematic review examined the association between drug use disorders and violence. We identified 18 eligible studies from 5 countries, with 591,411 individuals meeting diagnostic criteria for drug use disorders. There were 2 main findings. First, we found that individuals with a diagnosed drug use disorder have a 4- to 10-fold higher risk of perpetrating violence compared with general population or individuals without the drug use disorder being studied. All of the examined categories of drug use disorders—including cannabis, hallucinogens, stimulants, opioids, and sedatives—were associated with elevated violence risks. Of the total of 37 included studies, we found increased risk of violence in 34 studies with confidence intervals that did not cross 1. To examine the population impact, the odds of violence perpetration need to be seen in the context of general population prevalence of these disorders—which varies from 52 cases (per 100,000) of hallucinogen use disorders to 353 cases (per 100,000) of opioid use disorders (12). Second, there was substantial heterogeneity between studies, which was partially explained by study design and the type of outcome. Violence risk in drug use disorders was lower in cohort than in case-control studies, and when intimate partner violence was the outcome rather than general violence.

### Implications

Although the odds of increased risk of violence in drug use disorders are not dissimilar to those in other neuropsychiatric conditions (72), their importance is greater from a public health perspective as drug use disorders are more prevalent than severe mental illnesses, such as schizophrenia or bipolar disorder. In addition, although drug use disorders are not more prevalent than disorders such as depression and anxiety, their risk of violence is usually higher (67, 73). Therefore, drug use disorders have greater population impact when taking into account both prevalence and relative risk. This underscores the importance of treating drug use disorders as part of any public health approach to violence prevention. Notably, long-term methadone maintenance programs and behavioral treatments can reduce crime (74). In addition, there are studies that demonstrate reduced crime after drug treatment (e.g., opioid maintenance treatment, methadone, buprenorphine, and naltrexone) and nonmedical treatment (e.g., therapeutic communities, drug courts), among individuals using cocaine (75) and opioids (76–79), as well as with general drug use disorders (80–83). Moreover, prison-based interventions—such as therapeutic communities, opiate maintenance treatment, and pharmacotherapies for drug use disorders—are effective in reducing recidivism in prisoners (84–86). Despite this, most individuals with drug use disorders do not receive treatment. In the United States, among individuals with 12-month and lifetime drug use disorders, only 14% and 25% received treatment, respectively (87). Thus, more efforts should be made to improve accessibility of treatment for individuals with drug use disorders. Together, the treatability of drug use disorders, unmet needs, and risk of adverse outcomes present an opportunity to improve public health and safety.

A second implication, regarding study design, is that 2 aspects of design explained some of the between-study heterogeneity. Cohort studies had lower risk estimates than case-control investigations. This difference is likely because cohort studies are more likely to take into account the temporal sequence between drug use disorders and violent outcome. This allowed for a more accurate estimation of the associations than case-control studies. Future observational research should prioritize cohort designs to longitudinally follow individuals with drug use disorders and examine their violent outcomes. We also found that the association with intimate partner violence was less strong than with general violence. This might be because individuals with drug use disorders are less likely to have partners (87, 88) and those who have partners might present with less severe symptoms of drug use disorders (89).

### Strengths, limitations, and future directions

This review has several strengths. First, we included only studies that used validated diagnostic criteria to identify drug use disorders and excluded studies using self-report or other measures that might reflect short-term or recreational use. Second, we carefully explored heterogeneity using 2 methods (subgroup analyses and meta-regression). Third, we excluded studies examining drug use disorders and violent outcomes in selected samples such as offenders, cohorts with mental disorders, and individuals under treatment for drug use disorders, because not all individuals with drug use disorders are offenders or have other mental disorders, and the majority will not be subject to treatment. This likely increases the generalizability of our findings.

However, a number of limitations should be noted. First, all but one of the studies we included were conducted in high-income countries. We found an investigation from a middle-income country—Turkey—but no others, and none in Central Latin America, Tropical Latin America, and Southern sub-Saharan Africa, where violence is among the top 10 leading causes of disability-adjusted life-years (90). Many countries in these regions account for the majority of global drug manufacture, trafficking, and consumption (91, 92). Therefore, more research on the link in these settings is needed. A second limitation was that the amount of information on individual categories was not sufficient to draw definite conclusions about differences by drug class. We identified 3 studies (54, 59, 62) of sedative use disorder and 5 each for stimulant use disorder (54, 58, 59, 61, 62) and opioid use disorder (54, 59, 61, 62, 67). Furthermore, we found a limited literature on polydrug use, although it is common and linked to poorer treatment outcomes, social maladjustment, and overdose lethality (93–95). Future studies should investigate more carefully the different categories of drug use disorders, polydrug use, and links with novel psychoactive substances. Third, it is not possible to meta-analyze studies of selected populations because the effects of mediators cannot be modeled. Therefore, our findings are not necessarily risk estimates in specific subpopulations, such as prisoners or individuals who are participating in treatment programs. For example, our estimates might be overestimates given that we excluded studies of individuals under drug treatment, which could decrease risk of violence (96, 97). Fourth, we found links between hallucinogen use and violence in the general population, but there appears to be heterogeneity in their associations by population. For example, in criminal justice populations, recent work has found decreased associations between hallucinogen use and repeated offending in substance-involved offenders under community corrections supervision (48), which is also reported among intimate partner violence perpetrators (47, 98). Among individuals with schizophrenia, there is an increased risk (99). Finally, due to lack of data, we identified only a few factors that might explain heterogeneity between studies. For example, we were not able to examine whether some factors moderate the link between drug use disorders and violence, such as being subjected to violence, comorbidity of other substance use disorders (including alcohol) and mental health conditions, time between onset of drug use and violent outcome, and other social determinants (including poverty and access to services). In addition, the heterogeneity analyses were based on different drug categories and limited by variations in primary study settings. The results should therefore be interpreted with caution and read in the context of implications for future research rather than clinical practice.

Moreover, some factors could be associated with, could mediate, or could modify links between drug misuse and violence. For instance, an umbrella review of 22 meta-analyses based on over 120,000 individuals has shown that a range of neuropsychiatric disorders—including schizophrenia, personality disorders, and bipolar disorders—and perpetration, being a witness, or being a victim of violence during childhood are linked to increased risk of violence (72), suggesting that all of these comorbidities can be confounders. In addition, individuals who are victims of violence might use drugs as a coping mechanism, and victimization itself might in turn lead to later violence (35–39). Therefore, more research accounting for these factors is necessary.

### Conclusions

This systematic review has synthesized evidence on associations between individual categories of drug use disorder and violent outcomes. The findings suggest that all categories of drug use disorder have an elevated risk of violence, and that study design and type of violent outcome partly explain variation in risk estimates between studies.
